# Pharmaceutical payments to Japanese certified hematologists: a retrospective analysis of personal payments from pharmaceutical companies between 2016 and 2019

**DOI:** 10.1038/s41408-022-00656-y

**Published:** 2022-04-07

**Authors:** Eiji Kusumi, Anju Murayama, Sae Kamamoto, Moe Kawashima, Makoto Yoshida, Hiroaki Saito, Toyoaki Sawano, Erika Yamashita, Tetsuya Tanimoto, Akihiko Ozaki

**Affiliations:** 1 Department of Internal Medicine, Navitas Clinic Shinjuku, Shinjuku-ku, Tokyo Japan; 2 Medical Governance Research Institute, Minato-ku, Tokyo Japan; 3 Tohoku University School of Medicine, Sendai City, Miyagi Japan; 4 Hamamatsu University School of Medicine, Hamamatsu, Shizuoka Japan; 5 Fukushima Medical University, Fukushima City, Fukushima Japan; 6 Department of Gastroenterology, Sendai Kosei Hospital, Sendai City, Miyagi Japan; 7 Department of Surgery, Jyoban Hospital of Tokiwa Foundation, Iwaki City, Fukushima Japan; 8 Department of Internal Medicine, Navitas Clinic, Tachikawa City, Tokyo Japan; 9 Department of Breast and Thyroid Surgery, Jyoban Hospital of Tokiwa Foundation, Iwaki City, Fukushima Japan

**Keywords:** Drug development, Medical ethics

**Dear Editor**,

Collaboration between healthcare professionals, patients, and pharmaceutical companies have helped development of novel drugs and a better understanding of diseases [1]. Meanwhile, proper management of conflicts of interest (COIs) between healthcare sectors and pharmaceutical companies has increasingly become imperative among all healthcare professionals, as it may jeopardize patient-centered care [2]. In the field of hematology, a wide variety of therapeutic strategies and novel therapies attracted considerable attention from pharmaceutical companies, resulting in intense marketing by pharmaceutical companies. However, little is known about the characteristics and trends of personal payments from pharmaceutical companies to board-certified hematologists in Japan.

Primary aims of this study were to elucidate the prevalence of board-certified hematologists receiving payments, the magnitude of the payments, and the payment trend in recent years. All hematology specialists board-certified by the Japanese Society of Hematology were included in this study. The Japanese Society of Hematology is the sole society in Japan authorized to certify hematology specialists. The names, affiliations, and addresses of all board-certified hematology specialists were disclosed and collected from the Japanese Society of Hematology webpage (http://www.jshem.or.jp/modules/senmoni/) on October 10, 2021. Payment data of all healthcare professionals regarding lecturing, writing, and consulting were collected for the period 2016–2019 from all 92 pharmaceutical companies belonging to the Japan Pharmaceutical Manufacturers Association (JPMA). The JPMA is a voluntary trade organization comprising of a majority of the Japanese pharmaceutical companies that produce brand-name drugs and the JPMA-affiliated pharmaceutical companies accounted for 80.8% of total pharmaceutical sales in Japan in 2018 [3]. Detailed definitions of each payment category were described in Supplementary Material 1. Then we scanned for specialist names and extracted the payment data from the payment database of board-certified hematology specialists. Descriptive analyses of payment values and the number of instances were performed per board-certified hematology specialist and per pharmaceutical company. Average and median payments, instances, and number of companies making payments per board-certified hematology specialist were calculated based only on the number of board-certified hematology specialists receiving payments each year. Payment concentration was assessed by the Gini index and proportion of board-certified hematology specialists with more than specific monetary amounts. To examine the payment trend from pharmaceutical companies to board-certified hematology specialists from 2016 to 2019, the population-averaged generalized estimating equation (GEE) negative binomial regression model for the trend of payment value and the linear GEE log linked with binomial distribution for the trend of numbers of specialists with payments were performed, using the panel data of the annual personal payment values in each specialist.

Japanese yen (¥) was converted into USD ($) using the 2019 average monthly exchange rate of ¥109.0 per $1. This study was approved and informed consent was waived by the Ethics Committee of the Medical Governance Research Institute. Detailed methodologies were described in Supplementary Material 2 and our previous studies.

We identified 4183 hematology specialists certified by the Japanese Society of Hematology as of October 10, 2021. Of the 4183 board-certified hematology specialists, 2706 (64.7%) received a total of $36,291,434 (¥3,955,766,292) corresponding to 47,863 instances from 71 (77.2%) pharmaceutical companies between 2016 and 2019 (Table 1). The payment instances and values to the board-certified hematology specialists occupied 3.2% and 3.6% of total instances and values paid to healthcare professionals by all 92 pharmaceutical companies in Japan, respectively. The board-certified hematology specialists with payments received personal payments worth $13,411 (standard deviation (SD): $34,856) on average and a median of $2,471 (interquartile range (IQR): $851‒$9,677) over the 4-year period. The average number of instances and companies over the 4 years were 17.7 (SD: 35.9) and 5.5 (SD: 5.2), respectively (Table 1). The Gini index for the 4-year payments was 0.856. Top 1%, 5%, 10%, and 25% of board-certified hematology specialists occupied 26.0% (95% confidence interval (CI): 23.1–28.9%), 61.0% (95% CI: 58.2–63.7%), 76.8% (95% CI: 74.8–78.8%), and 93.3% (95% CI: 92.6–94.0%) of total payments, respectively (Supplementary Material 3). Further, among the 4183 board-certified hematology specialists, 2642 (63.2%) accepted one or more payments between 2016 and 2019 for the compensation of providing a lecture at an educational event sponsored by the pharmaceutical companies, while 1071 (25.6%) received the reimbursement for consulting between 2016 and 2019. (Supplementary Material 4).

**Table 1:** Summary of personal payments from pharmaceutical companies to hematology specialists certified by the Japanese Society of Hematology between 2016 and 2019.

Variables	
Total	
Payment values, $ (%)^a^	36,291,434 (3.6)
Instances, *n* (%)^b^	47,863 (3.2)
Companies, *n* (%)^c^	71 (77.2)
Average per specialist (SD)	
Payment values, $	13,411 (34,856)
Instances, *n*	17.7 (35.9)
Companies, *n*	5.5 (5.2)
Median per specialist (IQR)	
Payment values, $	2471 (851‒9677)
Instances, *n*	5 (2‒17)
Companies, *n*	4 (2‒8)
Range	
Payment values, $	46‒528,038
Instances, *n*	0‒487
Companies, *n*	0‒32
Physicians with specific payments, *n* (%)	
Any payments	2706 (64.7)
Payments >$500	2392 (57.2)
Payments >$1,000	1947 (46.6)
Payments >$5,000	980 (23.4)
Payments >$10,000	666 (15.9)
Payments >$50,000	175 (4.2)
Payments >$100,000	78 (1.9)
Gini index	0.856
Category of payments	
Lecturing	
Payment value, $ (%)	29,951,526 (82.5)
Instances, *n* (%)	40,686 (85.0)
Consulting	
Payment value, $ (%)	4,890,255 (13.5)
Instances, *n* (%)	5302 (11.1)
Writing	
Payment value, $ (%)	1,398,729 (3.9)
Instances, *n* (%)	1816 (3.8)
Other	
Payment value, $ (%)	50,924 (0.1)
Instances, *n* (%)	59 (0.1)

^a^The percentage was calculated by dividing the total payment values made to the board-certified hematologists by the total payment values made to healthcare professionals between 2016 and 2019 by 92 pharmaceutical companies.

^b^The percentage was calculated by dividing the total payment instances made to the board-certified hematologists by the total payment instances made to healthcare professionals between 2016 and 2019 by 92 pharmaceutical companies.

^c^The percentage was calculated by dividing the number of pharmaceutical companies making payments to the board-certified hematologists between 2016 and 2019 by 92 pharmaceutical companies.

Regarding the payment trend of 71 companies the data of which were available throughout 4 years, the median payments per board-certified hematology specialist and the number of board-certified hematology specialists with payments increased from $1241 (IQR: $511‒$3442) and 1808 (43.2%) in 2016 to $1629 (IQR: $613‒$4839) and 1844 (44.1%) in 2019, respectively. The payment values and number of board-certified hematology specialists increased by 11.2% (95% CI: 9.1–13.4%) and 1.8% (95% CI: 0.6–3.0%) each year (Table 2).

**Table 2:** Trend of personal payments from pharmaceutical companies to the hematology specialists board-certified by the Japanese Society of Hematology between 2016 and 2019.

Variables	2016	2017	2018	2019	Relative yearly change ratio (95% CI)	*P* value	Four years combined total
All pharmaceutical companies							
Total payments, $ (¥)	7,700,346 (839,337,686)	8,266,798 (901,080,935)	10,045,073 (1,094,912,919)	10,279,218 (1,120,434,752)	‒	‒	35,949,597 (3,955,766,292)
Average payments (SD), $	4259 (9291)	4918 (10,677)	5306 (11,403)	5574 (11,688)	1.112 (1.091‒1.134)	<0.001	13,411 (34,856)
Median payments (IQR), $	1241 (511‒3442)	1343 (525‒4087)	1504 (525‒4628)	1629 (613‒4839)			2471 (851‒9677)
Payment range, $	46‒106,834	46‒167,828	46‒148,942	52‒140,947	‒	‒	46‒528,038
Physicians with specific payments, *n* (%)							
Any payments	1808 (43.2)	1681 (40.2)	1893 (45.3)	1844 (44.1)	1.018 (1.006‒1.030)	0.003	2706 (64.7)
Payments >$500	1444 (34.5)	1373 (32.8)	1577 (37.7)	1570 (37.5)	1.040 (1.027‒1.054)	<0.001	2374 (56.8)
Payments >$1000	1024 (24.5)	998 (23.9)	1178 (28.2)	1159 (27.7)	1.055 (1.039‒1.071)	<0.001	1945 (46.5)
Payments >$5000	331 (7.9)	360 (8.6)	454 (10.9)	446 (10.7)	1.117 (1.088‒1.146)	<0.001	975 (23.3)
Payments >$10,000	187 (4.5)	208 (5.0)	239 (5.7)	253 (6.0)	1.109 (1.071‒1.149)	<0.001	660 (15.8)
Payments >$50,000	15 (0.36)	20 (0.5)	26 (0.6)	24 (0.57)	1.169 (1.033‒1.323)	0.014	172 (4.1)
Payments >$100,000	2 (0.048)	2 (0.048)	4 (0.096)	5 (0.12)	1.420 (0.940‒2.147)	0.096	75 (1.8)
Gini index	0.876	0.885	0.870	0.872	‒	‒	0.856
Pharmaceutical companies with 4-years payment data^a^							
Total payments, $ (¥)	7,700,346 (838,940,102)	8,266,798 (884,814,326)	10,045,073 (1,091,772,525)	10,142,157 (11,05,495,102)	‒	‒	35,972,679 (3,921,022,055)
Average payments (SD), $	4259 (9291)	4918 (10,677)	5306 (11,403)	5536 (11,557)	1.112 (1.091‒1.134)	<0.001	13,333 (34,685)
Median payments (IQR), $	1241 (511‒3442)	1343 (525‒4087)	1504 (523‒4628)	1633 (613‒4760)			2470 (851‒9500)
Payment range, $	46‒106,834	46‒167,828	0‒148,942	0‒137,882	‒	‒	46‒528,038
Physicians with specific payments, *n* (%)							
Any payments	1808 (43.2)	1681 (40.2)	1893 (45.3)	1844 (44.1)	1.018 (1.006‒1.030)	0.003	2698 (64.5)
Payments >$500	1444 (34.5)	1373 (32.8)	1577 (37.7)	1570 (37.5)	1.040 (1.027‒1.054)	<0.001	2383 (57.0)
Payments >$1000	1024 (24.5)	998 (23.9)	1178 (28.2)	1159 (27.7)	1.055 (1.039‒1.071)	<0.001	1938 (46.3)
Payments >$5000	331 (7.9)	360 (8.6)	454 (10.9)	446 (10.7)	1.117 (1.088‒1.146)	<0.001	973 (23.3)
Payments >$10,000	187 (4.5)	208 (4.8)	239 (5.7)	253 (6.0)	1.109 (1.071‒1.149)	<0.001	659 (15.8)
Payments >$50,000	15 (0.36)	20 (0.48)	26 (0.62)	24 (0.57)	1.169 (1.033‒1.323)	0.014	174 (4.2)
Payments >$100,000	2 (0.048)	2 (0.048)	4 (0.096)	5 (0.12)	1.420 (0.940‒2.147)	0.096	77 (1.8)
Gini index	0.876	0.885	0.870	0.872	‒	‒	0.857

*SD* Standard deviation, *IQR* interquartile range, *95% CI* 95% confidence interval.

^a^7 pharmaceutical companies were excluded because the companies did not disclose or we could not collect the 4 years payment data between 2016 and 2019.

There were 64 companies which made at least one payment during 4 years of the study period. Limiting to the payments from the 64 companies, both the average and median payment values constantly increased from $4259 (SD: $9291) and $1241 (IQR: $511‒$3442) to $5536 (SD: $11,557) and $1633 (IQR: $613‒$4760) between 2016 and 2019, respectively. The relative annual change rate for payments per specialist and number of specialists with payments also significantly increased by 11.2% (95% CI: 9.1–13.4%) and 1.8% (95% CI: 0.6–3.0%), respectively, each year.

Among 71 pharmaceutical companies making payments to board-certified hematology specialists, payments from the top 10 companies accounted for 70.8% of the total payments ($ 25,236,750) between 2016 and 2019 (Supplementary Material 5). The payment types for each of the top 10 paying companies are shown in Supplementary Material 6.

This study demonstrated that 3.6% ($36,291,434) of total personal payments concerning lecturing, consulting, and writing from all major pharmaceutical companies to healthcare professionals were distributed to board-certified hematology specialists, who accounted for 1.3% (4183 out of 327,210) of total physicians in Japan, according to the latest survey by the Japanese Ministry of Health, Labor and Welfare in 2018. To the best of our knowledge, this is the first study to assess the distribution and the trend of financial relationships between board-certified hematologists and pharmaceutical companies. Although our study could have limitations such as underreported payments due to the limited category of personal payments in Japan, there were important similarities and differences between our findings and those of the previous studies.

Previous studies in the United States demonstrated that 80.2% of hematologists and oncologists received general payments averaging $6,166 ($2055 in 1 year) between 2015 and 2017 [4]. Another study by Marshall et al. reported that 63.0% of medical oncologists, including pediatric hematologists/oncologists and hematologists/oncologists for adults in the United States, received $632 in median general payment in 2014 [5]. In the 6 years between 2014 and 2019, 84.6% (13,190 out of 15,585) of medical oncologists received $3,107 ($583‒$791 in a single year) in median general payment per physician [6]. Also, we previously evaluated pharmaceutical payments among Japanese board-certified oncologists, and found that 70.6% received a median of $1103 in annual personal payments [7]. Although the prevalence of the board-certified hematologists with payments were lower than those in other studies. Considering that this study covered only direct payments for lectures, consulting, and writing, the fact that 64.7% of board-certified hematologists in Japan received direct personal payments, mainly for giving lectures, is a notable finding. Further, our study showed that the median personal payments ranged from $1241 to $1629 were higher than those of medical oncologists in the United States and Japan.

Meanwhile, in other specialties in the US, the median total payments per physician were $88 among pediatricians in 2014 [8]; $194 among psychiatrists between 2016 and 2017 [9]; $145‒$184 in annual median payments among nephrologists [10]; $298 among dermatologists in 2014 [11]; $638 among ophthalmologists between 2013 and 2017 [12]; $1453 among cardiologists in median 3-year general payments between 2014 and 2016 [13]; $2818 among rheumatologists between 2014 and 2019 [14]; and $407 among neurologists in 2015 [15]. Comparing with these findings, the Japanese board-certified hematologists had similar or greater financial relationships with pharmaceutical companies than other specialists in the United States and Japan.

Further, regarding the trend of payments, we found that the personal payments increased significantly every year, with 11.2% yearly increase in the payments per board-certified hematology specialist. This trend of increasing payments to physicians was also observed among oncologists in the United States. Marshall et al. found that the total and annual average payment value per physician declined by −1.7% and −0.6%, respectively, since the launch of the US Open Payment Database in 2013. However, pharmaceutical companies increasingly prioritized the payments to board-certified hematologists and oncologists, with a 4.9% and 1.7% annual increase in total value and average payments. Furthermore, although the disclosure of personal payments from pharmaceutical companies to healthcare professionals and healthcare organizations was intended to curb the financial relationships, our findings indicate that disclosure itself did not sufficiently decrease the financial relationships between pharmaceutical companies and hematology board-certified specialists in Japan, as corroborated by previous studies in the United States.

The top 10 companies expanded their indications in the field of hematology, ranging from one to 11 new indications per company (Supplementary Material 7). While the payment from Celgene, the largest paying company, remained stable, four companies, namely, Takeda Pharmaceutical, Chugai Pharmaceutical, Janssen Pharmaceutical, and Novartis Pharma, remarkably increased their payments to the board-certified hematology specialists between 2016 and 2019. As a drug target, multiple myeloma accounted for the largest proportion (17 out of 52 indications), which may explain the recent trend of increasing payments.

This study has several limitations. First, our payment database was constructed by manually collecting payment data from 92 pharmaceutical companies. Despite careful and repeated checks, the inclusion of errors by our study team and pharmaceutical companies reporting data could not be ruled out. Additionally, other types of indirect payments, such as meals and beverages, travel, accommodations, research donations, and stock ownerships, were not disclosed with individual names of recipients by pharmaceutical companies in Japan. Thus, this study underreports the prevalence and magnitude of whole financial relationships. However, considering the payments for lecturing, writing, and consulting are directly paid to the healthcare professionals for the compensation of their work, this study highlights the magnitude and prevalence of direct financial relationships between pharmaceutical companies and Japanese board-certified hematologists. Third, this study included all board-certified hematologists as of October 2021, as the society did not disclose the list of board-certified hematologists for previous years, and there would have been some range of errors for our calculations. Still, this study generally assessed the magnitude and trends of the financial relationships over the past years.

In conclusion, this study found that majority of Japanese board-certified hematology specialists received personal payments as the reimbursement for lecturing, consulting, and writing from pharmaceutical companies. These personal payments from pharmaceutical companies were increasingly more prevalent and greater among Japanese board-certified hematology specialists.

## Supplementary information

Supplemental Material 1–7

Supplemental Material8_Dataset
