# Supplementary material for: Pharmaceutical payments to Japanese certified hematologists: a retrospective analysis of personal payments from pharmaceutical companies between 2016 and 2019
Source: Blood Cancer J. 2022 Apr 7;12(4):54. doi: 10.1038/s41408-022-00656-y (PMC8989935; doi:10.1038/s41408-022-00656-y)
Supplement: Supplementary file 1 — Supplemental Material 1–7 [file 41408_2022_656_MOESM1_ESM.docx]

Supplemental Material 1. Definitions of each payment category

| Payment category | Definition |
| --- | --- |
| Lecturing | Compensation for speaking and chairing in company-run educational events.  This payment category does not include a payment made to a participant of the company-run educational events. |
| Writing | Compensation for writing and supervising articles published by companies. |
| Consulting | Compensation for helping promote a specific medical product on the market and compensation for outsourcing work not applicable to the lecturing and the writing payment. |

Supplemental Material 2. Methodology

Certification of hematologists by the Japanese Society of Hematology

The Japanese Society of Hematology was established in 1937 and is the most prestigious and largest medical professional society in hematology in Japan, with 7,744 members as of October 8, 2021. The Japanese Society of Hematology is the sole society in Japan authorized to certify hematology specialists. As of October 2021, to be certified as a hematology specialist, physicians are required to meet all of the following six requirements: (1) submission of clinical records for 15 inpatients as an attending physician, including at least three cases of erythrocyte disease, three cases of leukocyte disease, two cases of thrombosis and hemostasis, and one case of immunology and blood transfusion; (2) must be listed as the first author of at least two academic articles or conference presentations; (3) must have completed at least six years of clinical practice training after having acquired a medical license and at least three years of specialized training in hematology at an institution accredited by the Japanese Society of Hematology; (4) must have a board certification in internal medicine issued by the Japanese Society of Internal Medicine or in pediatrics issued by the Japan Pediatric Society; (5) must be a registered member of the Japanese Society of Hematology for at least three years; and (6) must clear the written examination for the hematology specialists conducted by the Japanese Society of Hematology.

Data collection

The names, affiliations, and addresses of all board-certified hematology specialists were disclosed and collected from the Japanese Society of Hematology webpage on October 10, 2021. Payment data of all healthcare professionals and healthcare organizations regarding lecturing, writing, and consulting were collected for the period 2016-2019 from 92 pharmaceutical companies belonging to the JPMA. After collecting the payment data, a single unified database was developed, as described previously(1, 2). JPMA requires the member companies to disclose only the payment concerning lecturing, writing, and consulting, along with the individuals’ names and affiliations; therefore, these payment categories could be analyzed on an individual specialist basis. Personal payments, such as those for lecturing, writing, and consulting, are directly and widely paid to specialists by pharmaceutical companies.(2-6) Considering the nature of payments, only the personal payments concerning lecturing, writing, and consulting were included in this study.

We scanned for board-certified hematology specialist names in the payment database and extracted the payment data from the payment database of hematology specialists. The extracted data included the recipient names, affiliations, monetary amount, number of payment cases, payment category, and names of the respective pharmaceutical companies. To remove payment data of different persons with similar names in the database, we checked and compared the affiliations, affiliation addresses, and recipient specialties between the data from the Japanese Society of Hematology and the pharmaceutical companies For payments to board-certified hematology specialists whose affiliations reported by the pharmaceutical company differed from those reported by the society, we manually searched the name of the board-certified hematology specialists on the internet and collated other data from official institutional webpages and other sources to verify their identity. We also excluded payments that could not be verified from the analysis. The detailed procedure has been previously described(1, 2).

Furthermore, the lists of drugs newly approved for hematological diseases between 2015 and 2020 were collected from the webpage of the Pharmaceuticals and Medical Devices Agency, which is the official agency for reviewing drugs in Japan.

Analysis

Descriptive analyses of payment values and the number of cases were performed per board-certified hematology specialist and per pharmaceutical company. Average and median payments, cases, and number of companies making payments per board-certified hematology specialist were calculated based only on the number of specialists receiving payments each year, as in other studies.(4, 5, 7, 8) As several companies such as Shire Japan and Baxalta did not disclose the number of payment cases, the payments from these companies were excluded from the analysis of the number of cases, while the payment values were included in the analysis. To examine the concentration of payments to individual board-certified hematology specialists, the Gini index and shares of the value of payments held by the top 1%, 5%, 10%, and 25% of board-certified hematology specialists were calculated. The Gini index ranges from 0 to 1, and the greater the Gini index, the greater the disparity in the distribution of payments on a specialist basis, as mentioned previously.(1, 9) Payment distribution was also examined geographically.

Further, to examine the trend of payments from pharmaceutical companies to board-certified hematology specialists from 2016 to 2019, the population-averaged generalized estimating equation negative binomial regression model for the trend of payment value and the linear generalized estimating equation model log linked with binomial distribution for the trend of numbers of board-certified hematology specialists with payments were performed, using the panel data of the personal payments for each board-certified hematology specialist between 2016 and 2019. The relative ratios of the average annual increase in payments per board-certified hematology specialist to the number of board-certified hematology specialists with payments were used to report the results.

The year of payments was set as an independent variable, and the proportion of physicians receiving payments, number of payments, and payment values were set as dependent variables.(7, 10, 11) As several pharmaceutical companies disaffiliated from the JPMA and newly joined the JPMA, among all 92 companies, there were 18 companies without payment data over the four years. Thus, the average and median payments for each year and the trend of payments were calculated based on payments from all 92 companies and 74 companies with payment data for the four years between 2016 and 2019.

References

1. Murayama A, Ozaki A, Saito H, Sawano T, Shimada Y, Yamamoto K, et al. Pharmaceutical company payments to dermatology Clinical Practice Guideline authors in Japan. PLoS One. 2020;15(10):e0239610.

2. Ozaki A, Saito H, Onoue Y, Sawano T, Shimada Y, Somekawa Y, et al. Pharmaceutical payments to certified oncology specialists in Japan in 2016: a retrospective observational cross-sectional analysis. BMJ Open. 2019;9(9):e028805.

3. Hartung DM, Johnston K, Cohen DM, Nguyen T, Deodhar A, Bourdette DN. Industry Payments to Physician Specialists Who Prescribe Repository Corticotropin. JAMA Network Open. 2018;1(2):e180482-e.

4. Tringale KR, Marshall D, Mackey TK, Connor M, Murphy JD, Hattangadi-Gluth JA. Types and Distribution of Payments From Industry to Physicians in 2015. JAMA. 2017;317(17):1774-84.

5. Feng H, Wu P, Leger M. Exploring the Industry-Dermatologist Financial Relationship: Insight From the Open Payment Data. JAMA Dermatol. 2016;152(12):1307-13.

6. Inoue K, Blumenthal DM, Elashoff D, Tsugawa Y. Association between physician characteristics and payments from industry in 2015-2017: observational study. BMJ Open. 2019;9(9):e031010.

7. Tarras ES, Marshall DC, Rosenzweig K, Korenstein D, Chimonas S. Trends in Industry Payments to Medical Oncologists in the United States Since the Inception of the Open Payments Program, 2014 to 2019. JAMA Oncology. 2021;7(3):440-4.

8. Tringale KR, Hattangadi-Gluth JA. Types and Distributions of Biomedical Industry Payments to Men and Women Physicians by Specialty, 2015. JAMA Intern Med. 2018;178(3):421-3.

9. Yamamoto K, Murayama A, Ozaki A, Saito H, Sawano T, Tanimoto T. Financial conflicts of interest between pharmaceutical companies and the authors of urology clinical practice guidelines in Japan. Int Urogynecol J. 2021;32(2):443-51.

10. Marshall DC, Tarras ES, Rosenzweig K, Yom SS, Hattangadi-Gluth J, Murphy J, et al. Trends in Financial Relationships Between Industry and Radiation Oncologists Versus Other Physicians in the United States from 2014 to 2018. International Journal of Radiation Oncology*Biology*Physics. 2021;109(1):15-25.

11. Marshall DC, Tarras ES, Rosenzweig K, Korenstein D, Chimonas S. Trends in Industry Payments to Physicians in the United States From 2014 to 2018. JAMA. 2020;324(17):1785-8.

Supplemental Material 3. Concentration of payment to individual board-certified hematology specialists


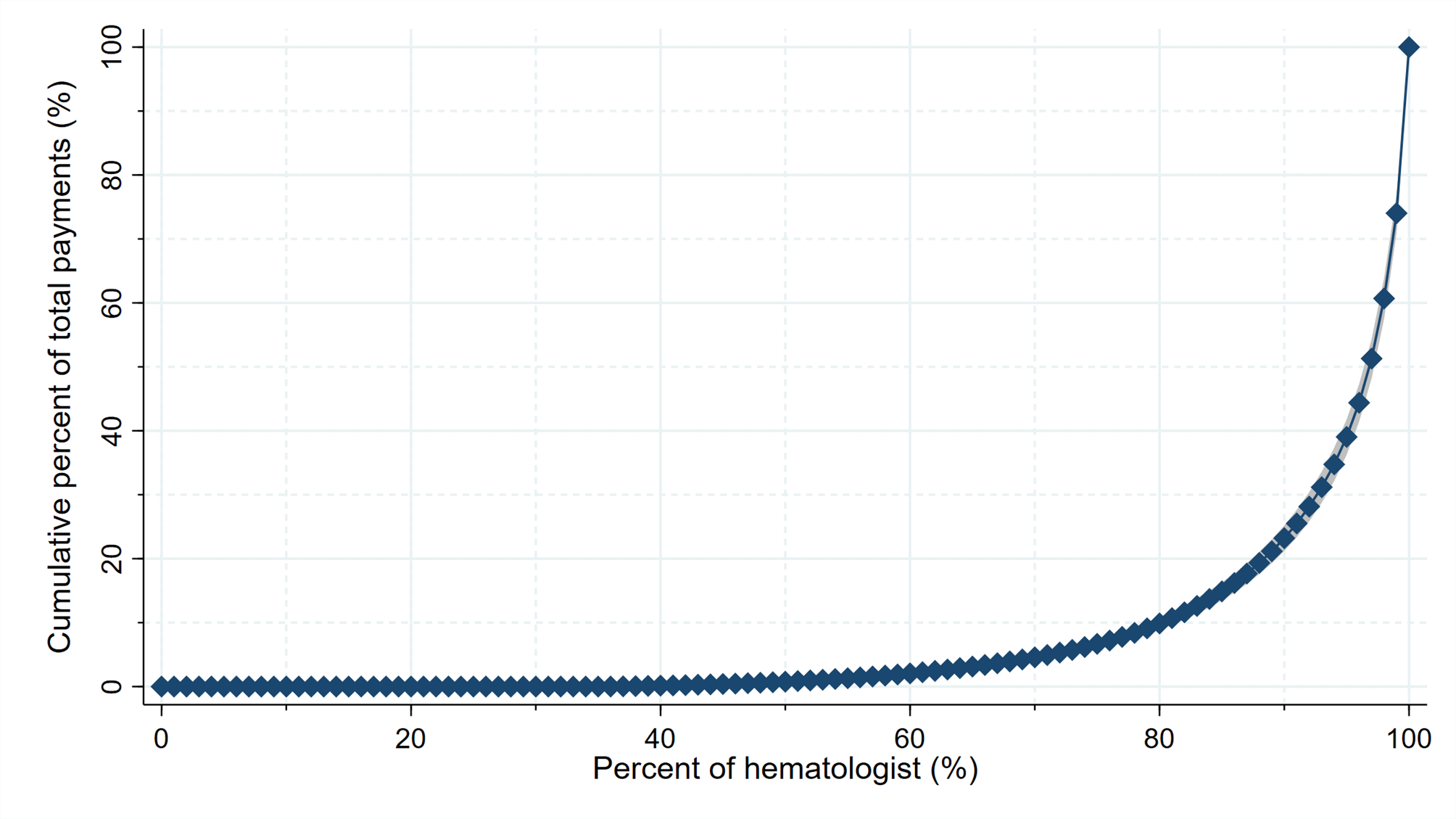


Supplemental Material 4. Number of the board-certified hematology specialists with payments by payment categories


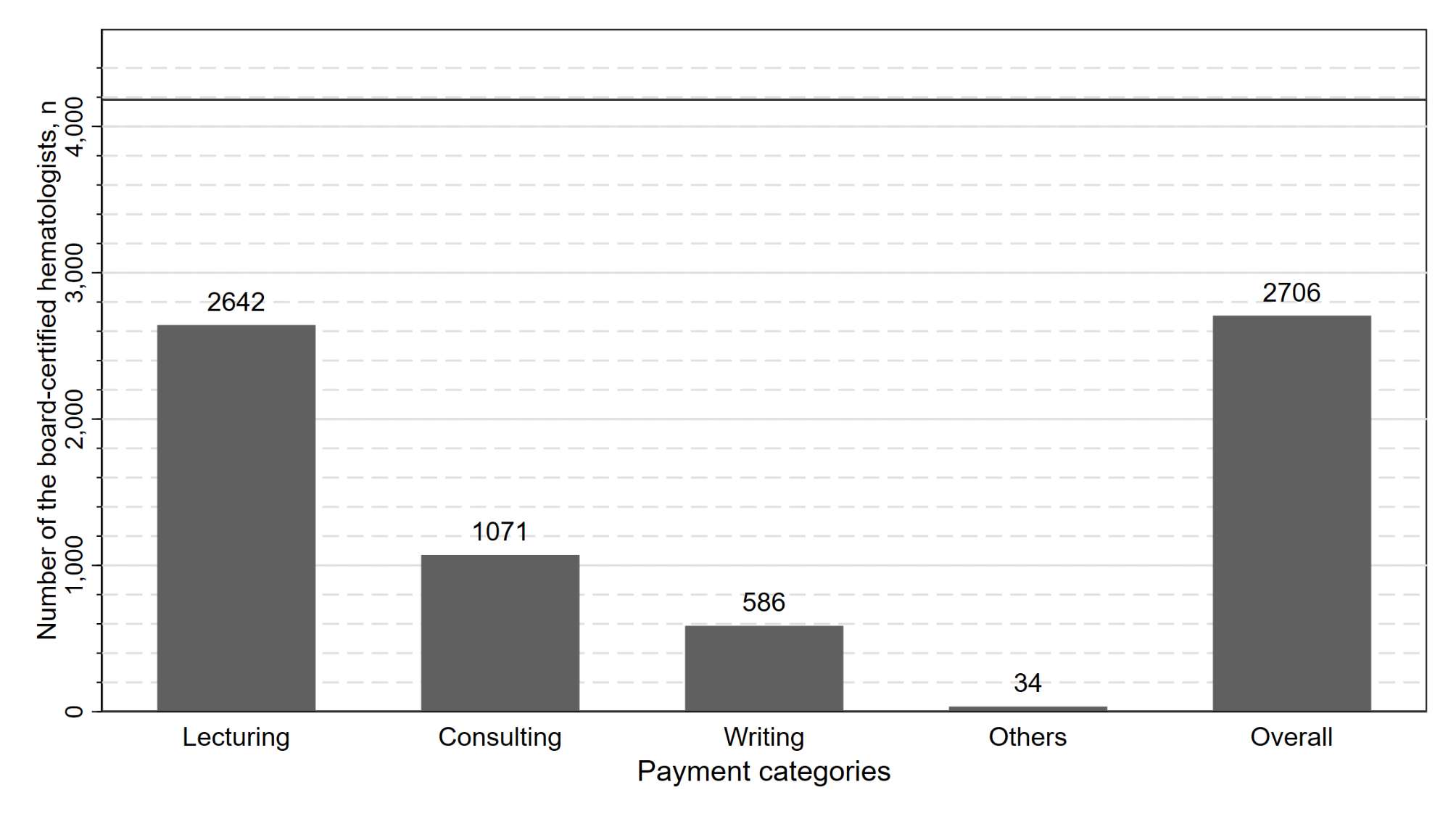


Supplemental Material 5. Total payments from top 10 largest paying pharmaceutical companies between 2016 and 2019


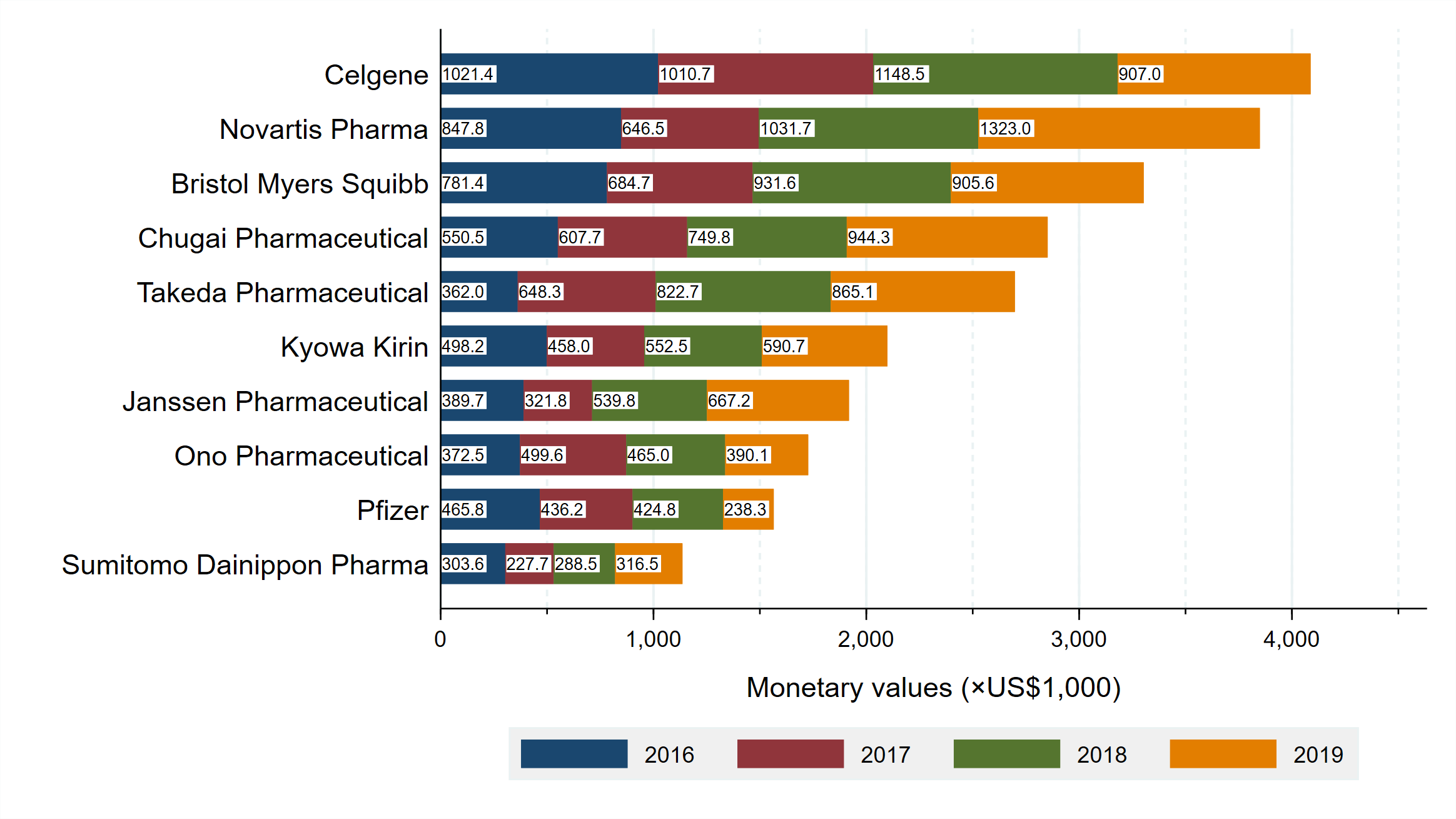


Supplemental Material 6. Category and distribution of payments by top 10 largest paying pharmaceutical companies


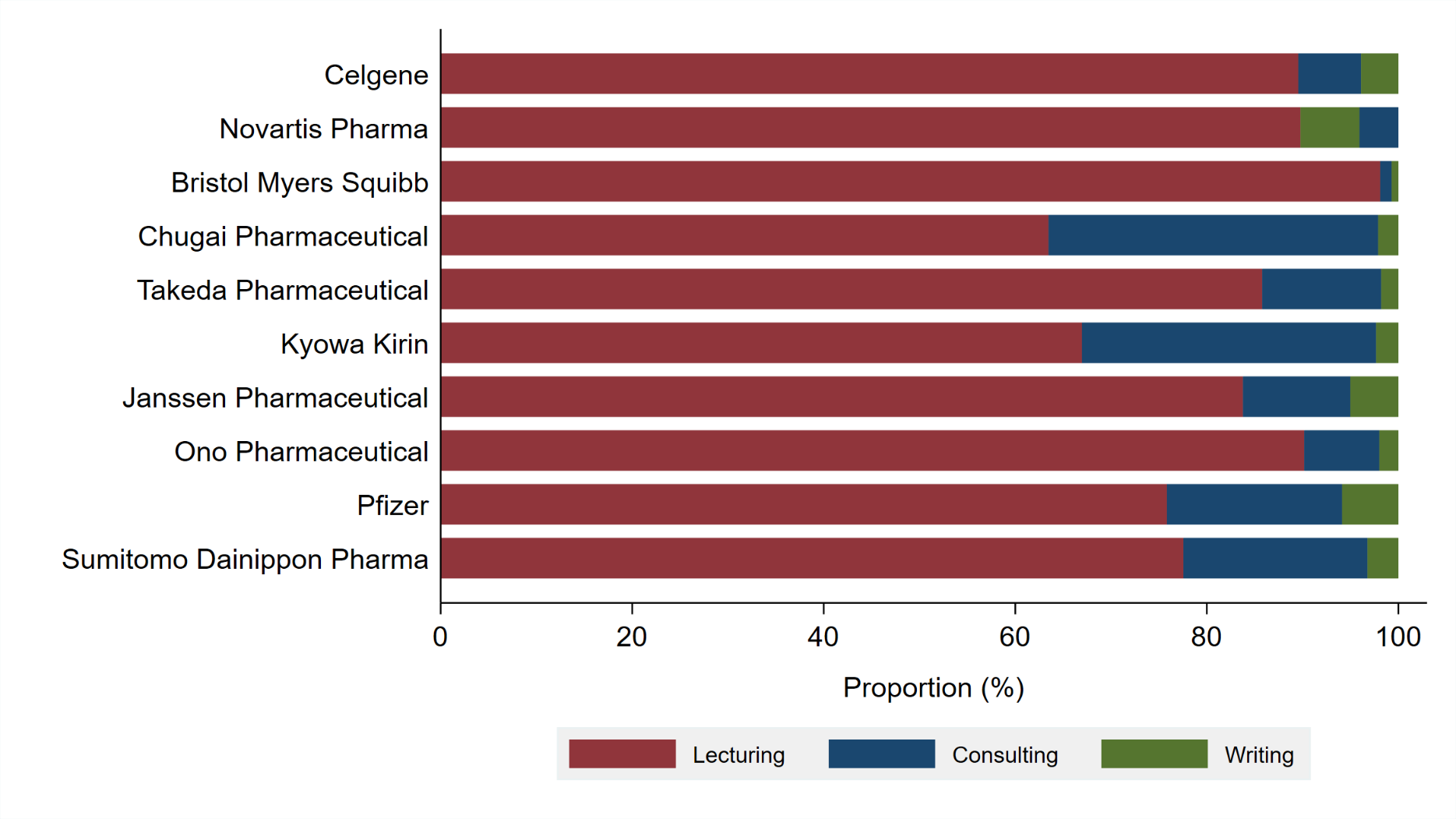


Supplemental Material 7. Drugs manufactured by the top 10 large-paying companies with new or additional indications for hematological disease between 2015 and 2020 in Japan

| Company | General name | Brand name | Approval date | Category of approval | Type of disease |
| --- | --- | --- | --- | --- | --- |
| Celgene | Lenalidomide hydrate | Revlimid | February 21, 2020 | Additional indication | Follicular lymphoma  Marginal zone lymphoma |
|  | Pomalidomide | Pomalyst | May 22, 2019 | Additional indication | Multiple myeloma |
|  | Romidepsin | Istodax | July 3, 2017 | New approval | Peripheral T-cell lymphoma |
|  | Lenalidomide hydrate | Revlimid | March 2, 2017 | Additional indication | Adult T-cell leukemia |
|  | Lenalidomide hydrate | Revlimid | December 21, 2015 | Additional indication | Multiple myeloma |
|  | Pomalidomide | Pomalyst | March 26, 2015 | New approval | Multiple myeloma |
| Novartis Pharma | Tisagenlecleucel | Kymriah | March 26, 2019 | New approval | CD19-positive B-cell acute lymphoblastic leukemia  CD19-positive diffuse large B-cell lymphoma |
|  | Nilotinib Hydrochloride Hydrate | Tasigna | December 25, 2017 | Added indication | Chronic myelogenous leukemia |
|  | Eltrombopag olamine | Revolade | August 25, 2017 | Additional indication | Aplastic anemia |
|  | Cyclosporine | Neoral | August 25, 2017 | Additional indication | Aplastic anemia |
|  | Deferasirox | Jadenu | July 3, 2017 | New approval | Chronic iron overload |
|  | Ruxolitinib Phosphate | Jakavi | September 24, 2015 | Additional indication | Polycythemia vera |
|  | Panobinostat lactate | Farydak | July 3, 2015 | New approval | Multiple myeloma |
| Takeda Pharmaceutical | Vonicog alfa^a^ | Vonvendi | March 25, 2020 | New approval | Von willebrand disease |
|  | Ixazomibe citrate ester | Ninlaro | March 25, 2020 | Additional indication | Multiple myeloma |
|  | Brentuximab vedotin | Adcetris | September 21, 2018 | Additional indication | CD30-positive hodgkin lymphoma |
|  | Rurioctocog alfa pegol^b^ | Adynovate | November 30, 2017 | Additional indication | Congenital blood coagulation factor 8 deficiency |
|  | Ixazomibe citrate ester | Ninlaro | March 30, 2017 | New approval | Multiple myeloma |
|  | Prednisolone | Prednisolone | June 26, 2015 | Additional indication | Multiple myeloma |
| Bristol Myers Squibb | Elotuzumab | Empliciti | November 22, 2019 | Additional indication | Multiple myeloma |
|  | Elotuzumab | Empliciti | September 28, 2016 | New approval | Multiple myeloma |
| Chugai Pharmaceutical | Alectinib hydrochloride | Alecensa | February 21, 2020 | Additional indication | Anaplastic large cell lymphoma |
|  | Emicizumab | Hemlibra | December 21, 2018 | Additional indication | Congenital blood coagulation factor 8 deficiency with inhibitor against blood coagulation factor 8 |
|  | Emicizumab | Hemlibra | December 21, 2018 | Additional indication | Congenital blood coagulation factor 8 deficiency without inhibitor against blood coagulation factor 8 |
|  | Obinutuzumab | Gazyva | July 2, 2018 | New approval | CD20-positive follicular lymphoma |
|  | Emicizumab | Hemlibra | March 23, 2018 | New approval | Congenital blood coagulation factor 8 deficiency |
| Kyowa Kirin | Romiplostim | Romiplate | June 18, 2019 | Additional indication | Aplastic anemia |
|  | Mogamulizumab | Poteligeo | August 21, 2018 | Additional indication | CCR4-positive adult T-cell leukemia-lymphoma  CCR4-positive peripheral t-cell lymphoma |
|  | Antithrombin gamma | Acoalan | July 3, 2015 | New approval | Congenital antithrombin deficiency |
| Janssen Pharmaceutical | Daratumumab | Darzalex | November 27, 2020 | Additional indication | Multiple myeloma |
|  | Daratumumab | Darzalex | December 20, 2019 | Additional indication | Multiple myeloma |
|  | Daratumumab | Darzalex | August 22, 2019 | Additional indication | Multiple myeloma |
|  | Bortezomib | Velcade | August 22, 2019 | Additional indication | Multiple myeloma |
|  | Ibrutinib | Imbruvica | July 2, 2018 | Additional indication | Chronic lymphocytic leukemia |
|  | Bortezomib | Velcade | March23, 2018 | Additional indication | Primary macroglobulinemia  Lymphatic plasma cell lymphoma |
|  | Daratumumab | Darzalex | September 27, 2017 | New approval | Multiple myeloma |
|  | Ibrutinib | Imbruvica | December 2, 2016 | Additional indication | Mantle cell lymphoma |
|  | Ibrutinib | Imbruvica | March 28, 2016 | New approval | Chronic lymphocytic leukemia |
|  | Bortezomib | Velcade | June 26, 2015 | Additional indication | Mantle cell lymphoma |
| Ono Pharmaceutical | Nivolumab | Opdivo | September 25, 2020 | Additional indication | Classic Hodgkin lymphoma |
|  | Tirabrutinib hydrochloride | Velexbru | August 21, 2020 | Additional indication | Primary macroglobulinemia  Lymphatic plasma cell lymphoma |
|  | Carfilzomib | Kyprolis | November 22, 2019 | Additional indication | Multiple myeloma |
|  | Nivolumab | Opdivo | September 21, 2018 | New approval | Classic Hodgkin lymphoma |
|  | Nivolumab | Opdivo | August 21, 2018 | Additional indication | Classic Hodgkin lymphoma |
|  | Carfilzomib | Kyprolis | May 18, 2017 | Additional indication | Multiple myeloma |
|  | Nivolumab | Opdivo | December 2, 2016 | Additional indication | Classic Hodgkin lymphoma |
|  | Carfilzomib | Kyprolis | July 4, 2016 | New approval | Multiple myeloma |
| Pfizer | Bosutinib hydrate | Bosulif | June 29, 2020 | Additional indication | Chronic myelogenous leukemia |
|  | Rituximab | Rituximab biosimilar | September 20, 2019 | New approval | CD20-positive B-cell non-Hodgkin lymphoma  CD20-positive B-cell lymphoproliferative disorder |
|  | Inotuzumab ozogamicin | Besponsa | January 19, 2018 | New approval | CD22-positive acute lymphocytic leukemia |
|  | Voriconazole | Vfend | August 24, 2015 | Additional indication | Prevention of cryptosporidiosis for hematopoietic stem cell |
| Sumitomo Dainippon Pharma | Thiotepa | Rethio | March 25, 2020 | Additional indication | Malignant lymphoma |

^a^Vonicog alfa (Vonvendi) was developed by Shire Japan, but due to the merger between Takeda Pharmaceutical and Shire Japan in 2019, we included vonicog alfaa as a product from Takeda Pharmaceutical. ^b^Rurioctocog alfa pegol was developed by Baxalta, and due to the merger between Baxalta and Shire Japan in 2016 and between Takeda Pharmaceutical and Shire Japan in 2019, we included vonicog alfaa as a product from Takeda Pharmaceutical.

Supplemental Material 8. Anonymized raw payment data
